# Influence of polymeric additives on the cohesion and mechanical properties of calcium phosphate cements

**DOI:** 10.1007/s10856-016-5665-x

**Published:** 2016-01-19

**Authors:** Jie An, Joop G. C. Wolke, John A. Jansen, Sander C. G. Leeuwenburgh

**Affiliations:** Department of Biomaterials, Radboud University Medical Center, PO Box 9101, 6500 HB Nijmegen, The Netherlands

## Abstract

To expand the clinical applicability of calcium phosphate cements (CPCs) to load-bearing anatomical sites, the mechanical and setting properties of CPCs need to be improved. Specifically, organic additives need to be developed that can overcome the disintegration and brittleness of CPCs. Hence, we compared two conventional polymeric additives (i.e. carboxylmethylcellulose (CMC) and hyaluronan (HA)) with a novel organic additive that was designed to bind to calcium phosphate, i.e. hyaluronan–bisphosphonate (HABP). The unmodified cement used in this study consisted of a powder phase of α-tricalcium phosphate (α-TCP) and liquid phase of 4 % NaH_2_PO_4_·2H_2_O, while the modified cements were fabricated by adding 0.75 or 1.5 wt% of the polymeric additive to the cement. The cohesion of α-TCP was improved considerably by the addition of CMC and HABP. None of the additives improved the compression and bending strength of the cements, but the addition of 0.75 % HABP resulted into a significantly increased cement toughness as compared to the other experimental groups. The stimulatory effects of HABP on the cohesion and toughness of the cements is hypothesized to derive from the strong affinity between the polymer-grafted bisphosphonate ligands and the calcium ions in the cement matrix.

## Introduction

Calcium phosphates (CaPs) have been extensively applied in dentistry, orthopedics and reconstructive surgery due to their excellent bone response [1]. CaPs are commercially available as pre-fabricated blocks and granules, which are difficult to handle from a clinical point of view. For example, CaP granules can migrate or dislocate easily into the surrounding tissue [2, 3]. Consequently, calcium phosphate cements (CPCs) have been widely investigated in view of their favorable handling properties. The self-hardening capacity of CPCs provides the possibility to fully adapt the bone substitute to the shape of the bone defect.

However, the risks associated with the use of CPCs as bone substitutes are related to the disintegration and the brittleness of CPCs. For example, premature disintegration can result in inflammatory responses [4]. In addition, these disintegrated cement particles may leak into the tissues surrounding the defect area, causing side effects such as nerve pain, venous and pulmonary embolism [5].

Concerning the brittleness of CPCs, it was shown previously that the flexural strength of CPC is low compared to bone, thereby limiting the applicability of CPCs to non-load-bearing anatomical sites [6]. To broaden the application of CPC to load-bearing applications such as spinal fusion [7], a toughened CPC with an increased fracture toughness needs to be developed. Several strategies can be used to overcome these drawbacks of CPCs. For example, by tuning the microstructural features of the precursor powders, the mechanical properties of the resulting CPCs can be optimized. Moreover, the chemical composition of the cement liquid and powder as well as the liquid to powder ratio of the substitute play an important role [8, 9]. However, the most common approach to reduce the brittleness of CPC for load-bearing applications involves the modification of the cement liquid with polymeric additives including discrete fibers or continuous networks [10, 11]. To this end, numerous polymeric additives have been explored, such as collagen, carboxylmethylcellulose (CMC) and hyaluronan (HA) [10, 12–14]. CMC is a commonly used additive in surgical applications due to its non-toxicity and biocompatibility [15]. The carboxyl group of this polymer provides the possibility to form electrostatic interactions with calcium ions in the CPC matrix [16]. Similarly, the carboxyl groups in HA allow for the formation of bonds with calcium ions in the CPC matrix. However, these electrostatic bonds are relatively weak and non-specific. Bisphosphonate (BP) drugs, on the other hand, display a very strong and specific affinity for calcium ions in the mineral phase of bone [17]. Recent insights on the interaction between bisphosphonates and precipitated nanocrystalline apatite surfaces indicated that the binding between bisphosphonate and calcium ions induces protonation and subsequent solubilization of orthophosphate ions from the apatite surface [18]. It was concluded that bisphosphonates not only complex with calcium ions, but also replace orthophosphate ions from apatitic surfaces, thereby ensuring a tight interaction with crystalline solids.

Previously, HA was derivatized with BP ligands to render HA calcium-binding. Previous results confirmed that this hyaluronan–bisphosphonate polymer (HABP) formed strong bonds with CaP nanoparticles both in vitro and in vivo [19]. Hence, we hypothesized that the covalent attachment of bisphosphonate groups to the polymer backbone of hyaluronan could improve the affinity of HA to the cement matrix, thereby improving the cohesion and mechanical properties of the resulting CPC. In order to evaluate the effects of this calcium-binding polymeric additive on the handling properties and mechanical properties of CPC, we compared this novel bisphosphonate-functionalized hyaluronan with two conventional, unmodified cohesion promoters, i.e., CMC and HA.

## Materials and methods

### Materials

Blanose sodium carboxyl methylcellulose (CMC, molecular weight 700 kDa, degree of substitution 0.88) was obtained from Brenntag (Brenntag Nederland BV, Rotterdam, the Netherlands) and sieved to remove any particles bigger than 106 µm, washed with 100 % isopropanol (analytical grade, Merck, Darmstadt, Germany) to remove potential microbiological contamination and dried at 90 °C overnight. Alpha-tricalcium phosphate (α-TCP) was provided by CAM Bioceramics BV (Leiden, The Netherlands). Sodium phosphate monobasic dihydrate (NaH_2_PO_4_·2H_2_O, Merck, Darmstadt, Germany) was used to form the basic liquid phase of the cement formulation. Hyaluronic acid (HA, molecular weight: 100–150 KDa) was purchased from Lifecore Biomedical (Chaska, The U.S.A),

### Methods

#### Synthesis and characterization of hyaluronan–bisphosphonate

Hyaluronan–thiol was synthesized according to a previously established protocol [19] and further functionalized with bisphosphonate via thiol–ene photopolymerization. To this end, various amounts of acrylated bisphosphonate (synthesized as described previously [20]) were added to 400 mg of hyaluronan–thiol in 80 ml degassed Milli-Q^®^ water in order to obtain bisphosphonate-to-thiol molar ratios of 4:1. Subsequently, 8 mg of Irgacure^®^ 2959 was added and the mixture was stirred for 10 min under ultraviolet light (36 W UV timer lamp, CNC international BV, The Netherlands). Thereafter, the mixture was dialyzed against 0.1 M NaCl at pH 3.5 (molecular weight cutoff of 3.5 kDa) and subsequently dialyzed twice against milli-Q water at pH 3.5. The solution was neutralized to pH 7.4 and lyophilized. The chemical composition of the polymer was confirmed using NMR [19].

#### CPC preparation

In total 7 CPC formulations were prepared at a fixed liquid to powder (L/P) ratio of 0.5 for all formulations. For the polymer-free CPC, 500 µl of a 4 w/v  % NaH_2_PO_4_·2H_2_O aqueous solution was added to 1 g of α-TCP powder inside a 2 ml plastic syringe (Kendall monoject, Gosport, UK), sealed with a closed tip and shaken for 25 s (Silamat^®^ mixing apparatus, Vivadent, Schaan, Liechtenstein). Formulations containing CMC were prepared by adding either 0.0075 g (0.75 wt%) or 0.015 g (1.5 wt%) of CMC powder to 0.9925 and 0.985 g of α-TCP powder, respectively, after which the powder mixture was shaken for 25 s in the Silamat^®^ mixing apparatus. Subsequently, 500 µl of a 4 % w/v NaH_2_PO_4_·2H_2_O solution was added to each syringe which was mixed vigorously for 25 s again using the Silamat^®^ mixing apparatus. For HA- and HABP-containing formulations, 0.0075 g (0.75 wt%) or 0.015 g (1.5 wt%) of each additive was dissolved in the liquid phase (4 % w/v aqueous solution of NaH_2_PO_4_·2H_2_O) prior to mixing 500 µl of the liquid phase to 1 g of α-TCP and shaking for 25 s in the Silamat mixer.

#### Setting time test

The initial and final setting time of the cement was assessed using Gillmore needles (ASTM, 1999). A bronze block was used as mould containing 6 holes (6 mm in diameter, 12 mm in height). The mould was placed in a water bath at body temperature (37 °C). All formulations were tested in threefold.

#### Cohesion test

The cohesion of the calcium phosphate paste was first evaluated qualitatively. Briefly, CPC paste was formed after mixing the contents for 25 s (Silamat^®^ Mixing apparatus; Vivadent). Subsequently, 1 g of each paste was injected into a 6 well culture plate containing 10 ml milli-Q water per well. All pastes were left for hardening in the well at room temperature for 4 h, after which the cohesion of the cements was recorded qualitatively using photographs.

Secondly, a method was developed to test the cohesion of the calcium phosphate paste quantitatively. To this end, a polytetrafluorethylene (PTFE) mold was designed containing a circular hole of 6 mm in diameter. After 25 s of mixing, seven composites were injected into seven separate PTFE molds, respectively. Subsequently, the surfaces of CPCs in the mold were smoothened and the molds containing the hardening CPCs were incubated in 10 ml of milli-Q water for 4 h at room temperature. The total handling time was fixed at 1 min including 25 s of mixing time. After 4 h of soaking, the PTFE mold was removed from the water, the supernatant was discarded, after which the sediment was freeze-dried and weighed. The quantitative evaluation of the wash-out was determined (n = 5) using the following equation:

1 $$Wash{\text{-}}out (\% ) = \frac{weight\,of\,sediment}{original\,weight\,of\,cement}\,*\,100\,\% $$

#### Mechanical properties

After mixing, the pastes were injected into a PTFE mold (cylinder shaped, diameter = 4.5 mm, height = 9 mm) to obtain cylindrical-shaped samples. Setting of the cement was performed within the mold at 100 % relative humidity for 24 h [21]. After soaking in Phosphate Buffered Saline (PBS) for 7 days, samples were placed in a tensile bench (858 MiniBionix2^®^, MTS Corp., Eden Prairie, MN, USA). The compressive strength of the samples was measured at a crosshead speed of 0.5 mm/min using a load cell of 2.5 kN.

Rectangular samples were produced (3 × 4 × 25 mm) in order to perform three-point bending tests [22]. After soaking the samples in PBS for 7 days, this three-point bending test was performed on a mechanical test bench at a cross-head speed of 0.5 mm/min using a load cell of 0.5 kN. Before testing, the samples were polished on one of their 4 × 25 mm^2^ surfaces using silicon carbide paper with grits of 1200. After fracture, the maximum load on the testing specimen was recorded and the bending strength of samples was calculated using Eq. 2 [23]:

2 $$Bending\, strength = \frac{{3P_{\hbox{max} } L}}{{2bh^{2} }}$$

where *P*_max_ is the maximum load on the load–displacement curve, L is the length of the support span, b is the specimen width and h is the specimen thickness (n = 5). Afterwards, the representative three-point bending load–displacement curves of both unmodified and polymer-modified CPC samples were recorded and the areas below the curves were divided by the specimen cross-section (bh) to obtain a quantitative measure for the toughness (in terms of work of fracture) of the samples. The test was stopped at a maximum crosshead displacement of 2 mm to allow for comparison between all experimental groups.

#### XRD

After the mechanical tests, solid samples were grinded to powder and powder X-ray diffraction (XRD, Philips, PW 3710, Almelo, the Netherlands) was applied to determine the crystal phases of the cement composites.

#### SEM

The fracture surfaces after the bending tests were collected for morphological analysis using scanning electron microscopy (SEM, JEOL6340F, Tokyo, Japan, operated at 10 kV and a working distance = 15 mm).

### Statistical analysis

Data were presented as mean ± standard deviation. Significant differences were determined using one-way analysis of variance (One-way ANOVA) followed by a Tukey post hoc test. Results were considered significant if *P* < 0.05. Calculations were performed using GraphPad Instat^®^ (GraphPad Software Inc., San Diego, CA, USA).

## Results

### Setting time of CPCs

**Fig. 1:**
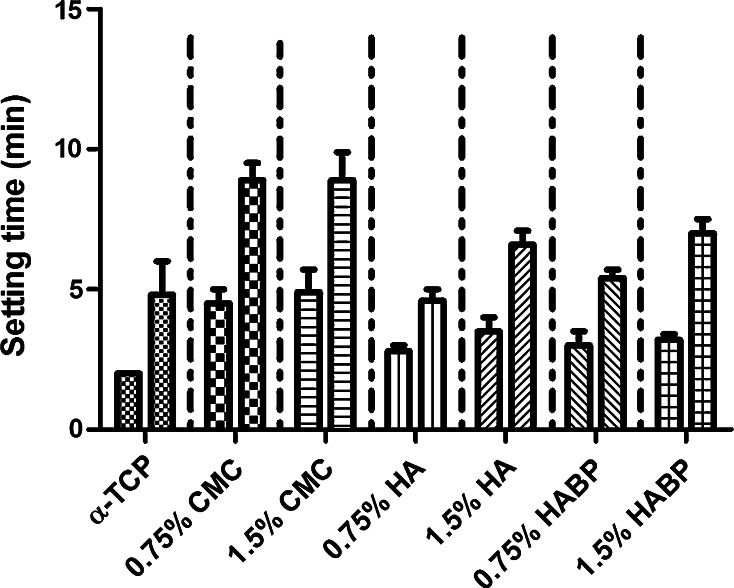
Initial (*left columns*) and final setting times (*right columns*) of CPCs containing different amounts and types of polymeric additives (n = 3)

The initial setting time of α-TCP was 2 min and the final setting time was 4.8 ± 1.2 min (Fig. 1). All polymeric additives delayed the initial setting time of the CPCs, but the addition of CMC resulted into the most pronounced delaying effect on the initial (4.5 ± 0.5 min for 0.75 % CMC and 4.9 ± 0.8 min for 1.5 % CMC) and final setting times (8.9 ± 0.6 min for 0.75 % CMC and 8.9 ± 1 min for 1.5 % CMC). The addition of HA, on the other hand, resulted into a minor delaying effect on the initial (2.8 ± 0.2 min for 0.75 % HA and 3.5 ± 0.5 min for 1.5 % HA) and final setting times (4.6 ± 0.4 min for 0.75 % HA and 6.6 ± 0.5 min for 1.5 % HA). The initial setting times of CPCs containing 0.75 % HABP and 1.5 % HABP groups were comparable (3 ± 0.5 min and 3.2 ± 0.2 min, respectively), whereas the final setting of the CPC containing the highest amount of HABP (1.5 %) was longer (7 ± 0.5 min) compared to the group with the lower amount (0.75 %) of HABP (5.4 ± 0.3 min).

### Cohesion

**Fig. 2:**
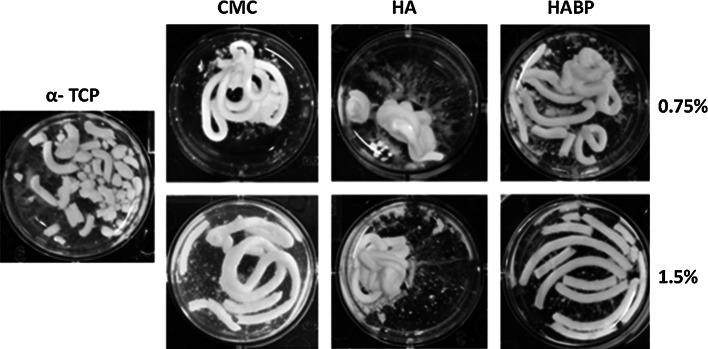
Disintegration of the cements after extrusion in water and 4 h of immersion

A qualitative impression of the cohesion of the cements is shown in Fig. 2. Monophasic α-TCP-based cement disintegrated into small pieces surrounded by a cloudy supernatant after 4 h of soaking. Cements containing 0.75 % CMC showed a stable and curved wire-like shape after injection. However, the shape of 1.5 % CMC group expanded during soaking resulting in an increased thickness of the wire-like shape and a cloudy supernatant. Both 0.75 % HA and 1.5 % HA formulations maintained their shape after extrusion of the CPC into water, without the presence of wire-like features that are characteristic for cohesive formulations. Incorporation of 0.75 % HABP into CPCs resulted into stable and straight wire-like shapes as well as minor particles, while the cement was extruded as discrete, straight wires upon incorporation of 1.5 % HABP into the cement.

**Fig. 3:**
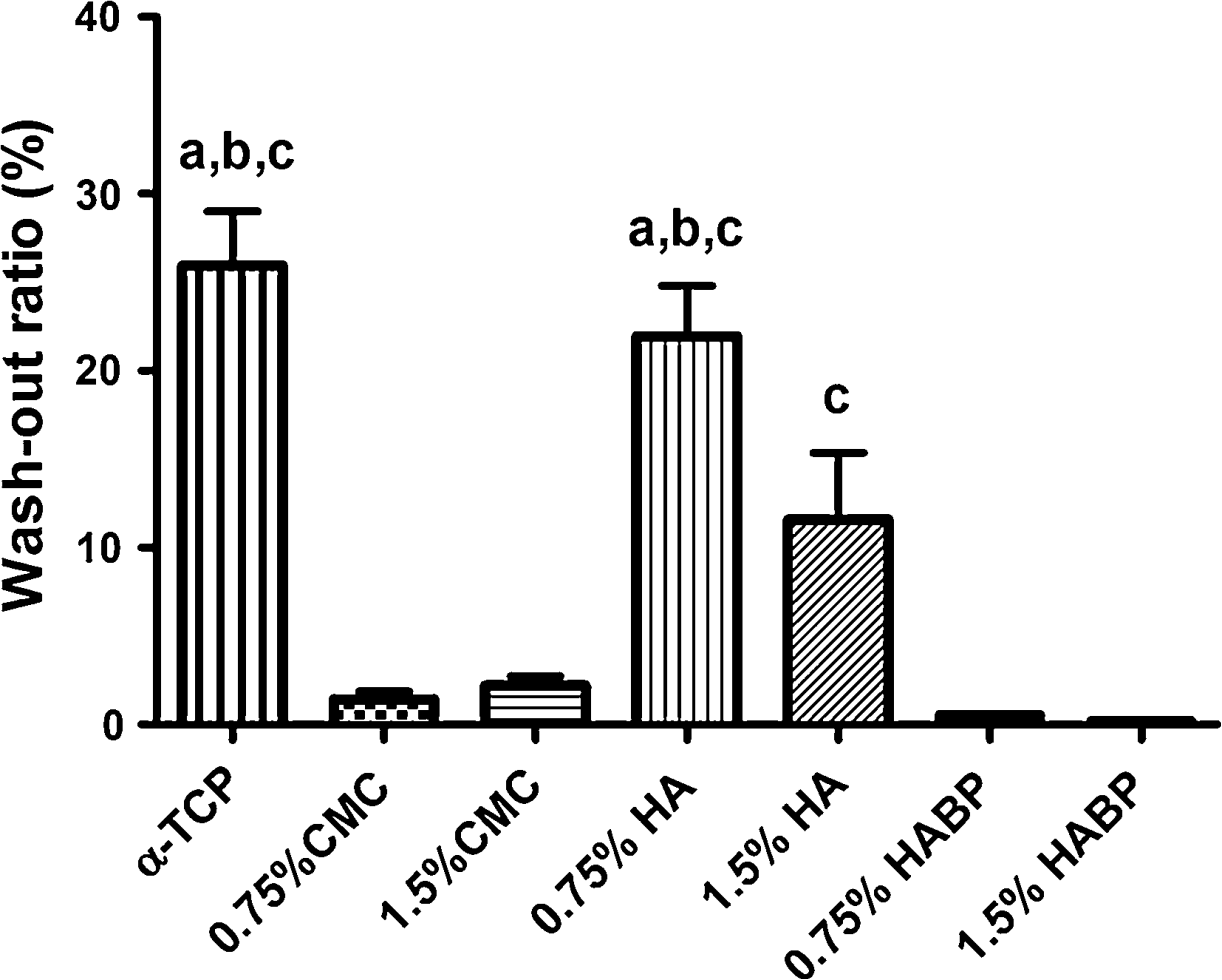
Wash-out ratio of α-TCP and polymer-containing composites after 4 h of immersion. **a** Significantly different compared to CMC-containing groups (*P* < 0.05), **b** different compared to 1.5 % HA (*P* < 0.05), **c** significantly different compared to HABP-containing groups (*P* < 0.05)

The quantitative evaluation of cohesion provided additional insight into the cohesion of the various formulations. All formulations showed a significantly reduced wash-out ratio compared to the unmodified cement control (Fig. 3). The addition of CMC and HABP resulted into strongly reduced wash-out ratios, whereas only a minor effect on the wash-out ratio was observed upon addition of unfunctionalized hyaluronan.

### Mechanical properties

**Fig. 4:**
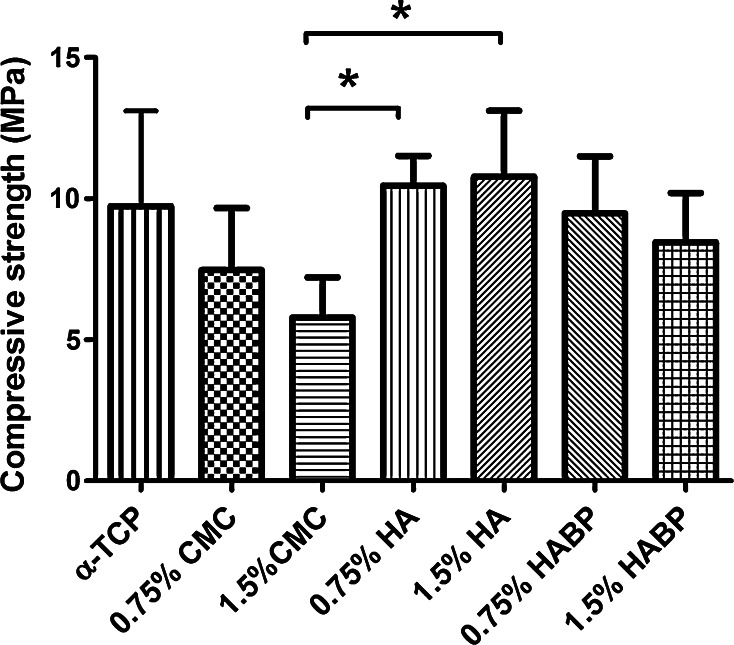
Compressive strength of samples after 7 days in PBS, significant differences were marked with an asterisk when *P* < 0.05

The compressive strength of α-TCP and CPC composites after 7 days of soaking in PBS is depicted in Fig. 4. After adding the polymeric additives, no differences were found in compressive strength compared to unmodified cements (10.5 ± 2.0 MPa). However, the compressive strength of cements containing 1.5 % CMC (5.8 ± 1.3 MPa) was significantly lower compared to the groups containing HA (0.75 % HA = 10.5 ± 0.9 MPa, 1.5 % HA = 10.8 ± 2.1 MPa).

**Fig. 5:**
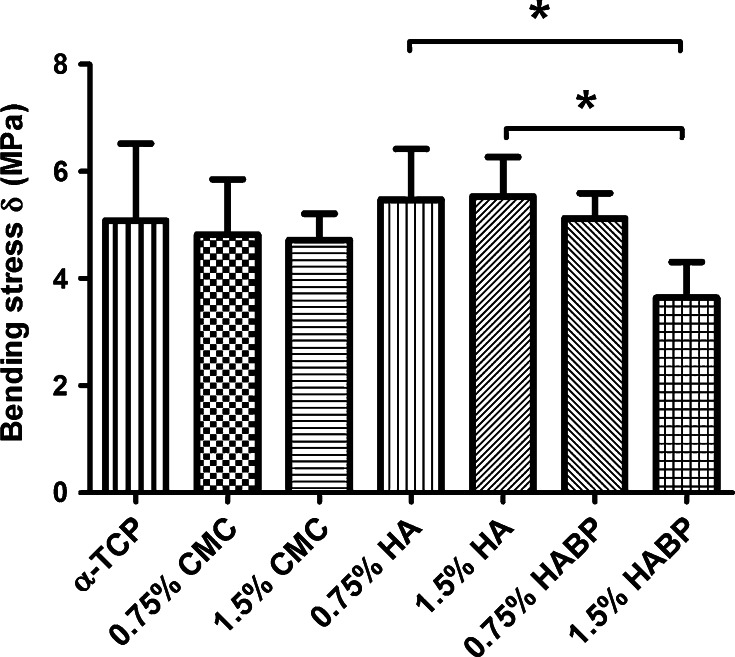
Bending strength of samples after 7 days in PBS, significant differences were marked with an asterisk when *P* < 0.05

The bending strength (Fig. 5) as obtained from the three-point bending test revealed no differences between unmodified and polymer-modified modified cements, whereas a significantly lower bending strength was obtained for cements containing 1.5 % HABP (3.6 ± 0.7 MPa) compared to cements containing bisphosphonate-free HA as additive (0.75 % HA = 5.5 ± 1 MPa, 1.5 % HA = 5.5 ± 0.7 MPa).

**Fig. 6:**
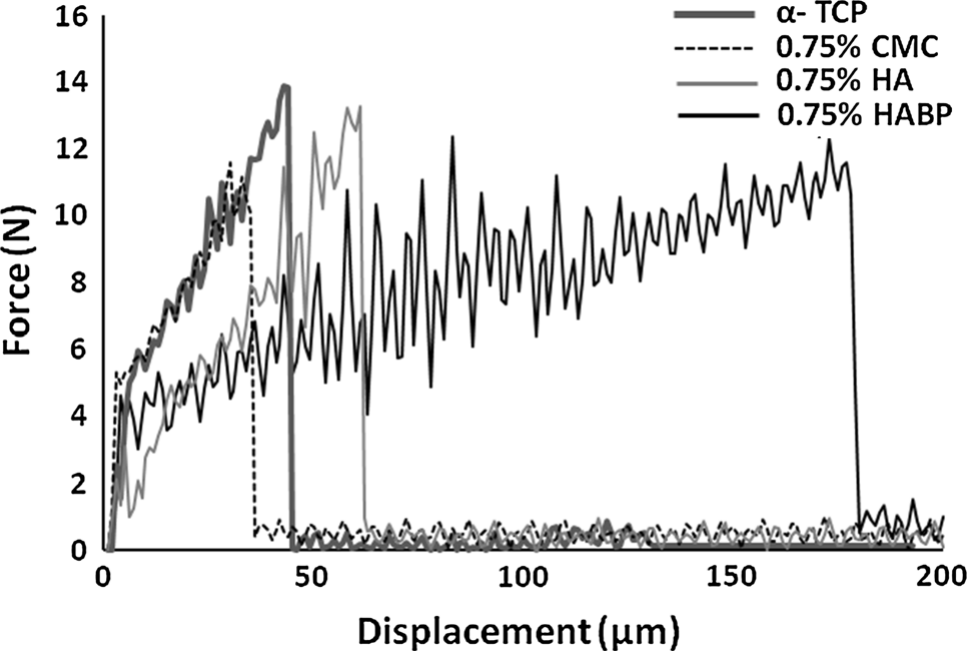
Representative three-point bending load–displacement curves of unmodified cements (α-TCP) and cements containing 0.75 % CMC, 0.75 % HA or 0.75 % HABP

**Fig. 7:**
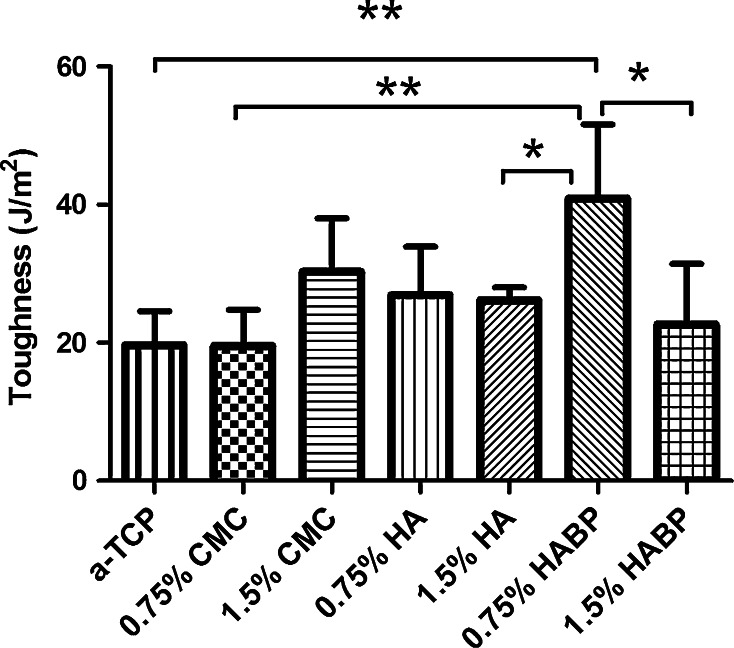
The toughness after the three-point bending test of unmodified cements (α-TCP) and cements containing various amounts and types of polymeric additives. ** indicates *P* < 0.01, * indicates *P* < 0.05

Figure 6 shows representative load–displacement curves of the three-bending tests for unmodified cements (α-TCP) and cements containing 0.75 % of CMC, HA or HABP. The curves indicated that unmodified CPC (α-TCP) fractured in a brittle manner after reaching the peak load of the material, after which the load-bearing capacity decreased abruptly. A similarly sharp decrease was observed for cements containing 0.75 % CMC or 0.75 % HA. However, cements containing 0.75 % of HABP displayed higher extensibility than the other groups. Figure 7 shows that the toughness for cements containing 0.75 % HABP (40.9 ± 10.8 J/m^2^) was significantly higher than unmodified cements (19.6 ± 5 J/m^2^, *P* < 0.001) or cements containing 0.75 % CMC (19.5 ± 5.3 J/m^2^, *P* < 0.001), 1.5 % HA (26.1 ± 1.92 J/m^2^, *P* < 0.05) or 1.5 % HABP (22.7 ± 8.8 J/m^2^, *P* < 0.05).

### XRD

**Fig. 8:**
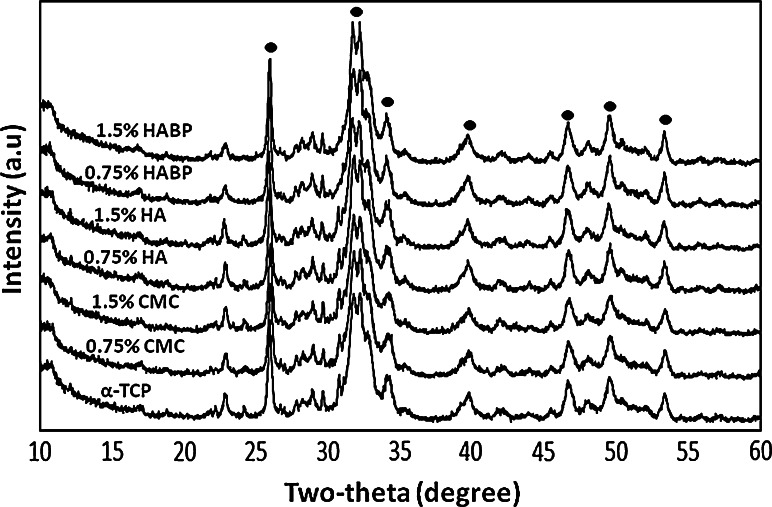
X-ray diffraction patterns after 7 days of incubation of unmodified cements (α-TCP) and cements containing 0.75 % CMC, 1.5 % CMC, 0.75 % HA, 1.5 % HA, 0.75 % HABP and 1.5 % HABP. Main apatitic reflections are indicated with black ovals

X-ray diffraction (XRD) patterns (Fig. 8) showed that all cements were converted to the apatite phase (main reflections indicated with black ovals) after 7 days of incubation. No differences were observed between the various experimental groups.

### SEM

**Fig. 9:**
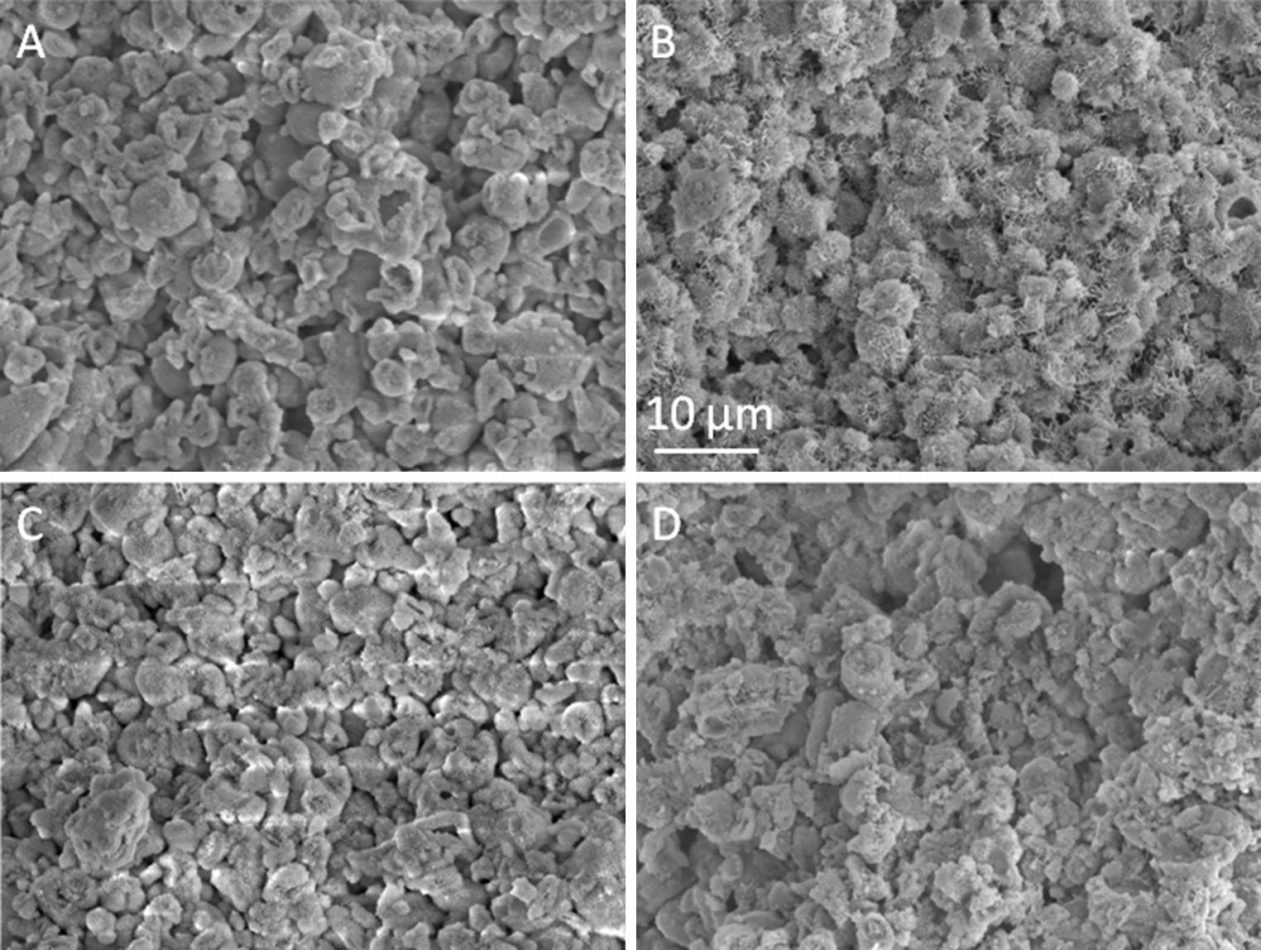
Scanning electron micrographs of the fracture surfaces of unmodified cements (**a**) or cements modified with 1.5 % CMC (**b**), 1.5 % HA (**c**) or 1.5 % HABP (**d**)

SEM micrographs of the fractured surfaces after three-point bend testing were analyzed and the representative images of α-TCP, 1.5 % CMC, 1.5 % HA and 1.5 % HABP are shown in Fig. 9. All fracture surfaces were rough and irregular with several micron-scale pores at the fracture surface. Sub-micron crystals were observed on the fracture surfaces of the cements containing 1.5 % CMC or 1.5 % HABP.

## Discussion

 The setting time of self-setting CPCs is a crucial parameter for their clinical applicability. Since the clinical wound area can only be closed after setting of the cement, the time required for this hardening process is very critical. For optimal clinical handling of CPCs, the suggested final setting time should be below 15 min [24]. The setting properties of cements have been modified by controlling the particle size of calcium phosphate precursor powders, adding a nucleating phase, or dissolving additives into the liquid phase that accelerate or inhibit the setting reaction [25]. The XRD analysis confirmed that after reacting with the liquid phase, α-TCP converted into hydroxyapatite. Previous reports indicated that organic additives can influence this transformation process [26–28], but none of the polymeric additives selected in the current study impaired the transformation from α-TCP to hydroxyapatite.

The setting time of CPCs increased from 2 to 5 min for the initial setting and from 6 to 10 min for the final setting. The addition of the lowest amount of HA (0.75 %) did not affect the setting of the CPCs, but the highest amount of HA (1.5 %) resulted into a delayed setting as well. This observation is in agreement with the research by Kai et al. [29] who observed that setting times increased with increasing amount of HA incorporation. Since both CMC and HA contain pendant carboxyl groups, we speculate that the more pronounced delaying effect of CMC on the setting of CPC was caused by physical factors such as a higher viscosity of CMC-containing solutions. Regarding the effect of free or conjugated bisphosphonates, it was shown by Panzavolta et al. that free alendronate (added to the cement at high concentrations of about 1 mM) delayed the initial and final setting times of CPCs to 10 and 33 min, respectively [30]. In the current study we also observed a delaying effect of bisphosphonate-functionalized HA on the setting properties of CPC, albeit to a much lower extent. This phenomenon can be explained by the fact that the bisphosphonate concentration in the study of Panzavolta et al. was much higher (1 mM) than in the current study (degree of bisphosphonate-for-carboxyl substitution of 8 % [20] at HABP contents of 0.75 and 1.5 wt% only). Consequently, the initial and final setting times of HABP-modified cements were still within the acceptable range for clinical handling and workability.

CPCs are materials designed to be implanted as a paste, which implies that the paste is in contact with blood or other body fluids upon surgical application. The capacity of CPCs to set in a fluid without disintegration into smaller fragments is often referred to as ‘cohesion’. Several approaches have been adopted to improve the cohesion of CPCs, such as lowering the liquid to powder ratio (L/P), decreasing the particle size of calcium phosphates, and replacing the liquid phase with a viscous polymeric solution [31, 32]. Here, we studied the addition of several organic additives as cohesion promoters to the cement formulation. Direct addition of CMC to the liquid phase of the cements resulted in highly viscous solutions, which compromised the injectability of the cements. Therefore, we added CMC to the powder phase (α-TCP) of the cement formulation. In this way, the reaction time between CMC and the liquid phase was controlled without compromising the injectability and cohesion of the cements. The wash-out ratio of α-TCP was decreased most effectively by the incorporation of CMC and HABP as cohesion promoters. CMC apparently immobilized the CPC particles, thereby improving the washout resistance of the cement [13]. HABP acted as an effective binder by forming electrostatic interactions between calcium ions in the CPC matrix and the calcium-binding bisphosphonate groups conjugated to the HA backbone.

Generally, the brittleness of CPCs limits the long-term performance and clinical applicability of CPCs. Recent studies indicated that the strength and toughness of the cements can be substantially improved by polymeric reinforcements, thereby providing the potential to facilitate applications in load-bearing skeletal sites [22, 33–35]. A large number of parameters affect the mechanical properties of these materials, such as the cement preparation and the self-setting reaction. Previous results demonstrated that the weak mechanical properties of CPC are mainly caused by the inherent high porosity, while the compressive strength of CPCs was increased considerably by lowering the porosity into more dense microstructures [9, 36, 37]. Generally, we observed that the coefficient of variation of both the compressive and bending strength of the cements was large, which is typical for ceramic materials due to the presence of surface defects and internal pores. We did not observe any positive effect of the selected polymeric reinforcements on the compression or bending strength, while the compression and bending strength of the CPCs were compromised by the addition of the highest amount (1.5 wt%) of CMC and HABP, respectively. From SEM observations we can conclude that sub-micron crystals were present at the fracture surfaces of CPCs containing 1.5 % CMC or HABP, which might have introduced nanoporosity into the cements that contributed to lower strength values. The only polymeric additive that effectively increased the cement toughness was HABP at a concentration of 0.75 wt%. Nevertheless, it can be concluded that the addition of the selected polymeric additives resulted into pronounced effects on cement setting and cohesion, but only marginal effects of cement strength and toughness.

## Conclusions

The effect of two conventional polymeric additives (i.e. CMC and HA) on the cohesion, setting and mechanical properties of calcium phosphate cements was compared to a novel organic additive that was designed to bind to calcium phosphate, i.e. hyaluronan–bisphosphonate (HABP). The cohesion of α-TCP was improved considerably by the addition of CMC and HABP. None of the additives improved the compression and bending strengths of the cements, but the addition of 0.75 % HABP resulted into a significantly increased cement toughness as compared to the other experimental groups.

## References

[1] Pili D, Tranquilli LP (2011). Biomaterials and bone. Aging Clin Exp Res..

[2] Wittkampf AR (1989). Fibrin glue as cement for HA-granules. J Craniomaxillofac Surg.

[3] Kent JN, Quinn JH, Zide MF, Guerra LR, Boyne PJ (1983). Alveolar ridge augmentation using nonresorbable hydroxylapatite with or without autogenous cancellous bone. J Oral Maxillofac Surg.

[4] Miyamoto Y, Ishikawa K, Fukao H, Sawada M, Nagayama M, Kon M (1995). In vivo setting behaviour of fast-setting calcium phosphate cement. Biomaterials.

[5] Krebs J, Aebli N, Goss BG, Sugiyama S, Bardyn T, Boecken I (2007). Cardiovascular changes after pulmonary embolism from injecting calcium phosphate cement. J Biomed Mater Res B Appl Biomater.

[6] Geffers M, Groll J, Gbureck U (2015). Reinforcement strategies for load-bearing calcium phosphate biocements. Materials.

[7] Tarsuslugil SM, O’Hara RM, Dunne NJ, Buchanan FJ, Orr JF, Barton DC (2013). Development of calcium phosphate cement for the augmentation of traumatically fractured porcine specimens using vertebroplasty. J Biomech.

[8] Kiyasu K, Takemasa R, Ikeuchi M, Tani T (2013). Differential blood contamination levels and powder–liquid ratios can affect the compressive strength of calcium phosphate cement (CPC): a study using a transpedicular vertebroplasty model. Eur Spine J.

[9] Ginebra M, Driessens F, Planell J (2004). Effect of the particle size on the micro and nanostructural features of a calcium phosphate cement: a kinetic analysis. Biomaterials.

[10] Moreau JL, Weir MD, Xu HH (2009). Self-setting collagen-calcium phosphate bone cement: mechanical and cellular properties. J Biomed Mater Res A..

[11] O’Hara RM, Orr JF, Buchanan FJ, Wilcox RK, Barton DC, Dunne NJ (2012). Development of a bovine collagen–apatitic calcium phosphate cement for potential fracture treatment through vertebroplasty. Acta Biomater.

[12] Rehberg S, Zwipp H, Rammelt S (2009). In vivo effects of modification of hydroxyapatite/collagen composites with and without chondroitin sulphate on bone remodeling in the sheep tibia. J Orthop Res..

[13] Cherng A, Takagi S, Chow L (1997). Effects of hydroxypropyl methylcellulose and other gelling agents on the handling properties of calcium phosphate cement. J Biomed Mater Res.

[14] Mark HF (1968). Encyclopedia of polymer science and technology: plastics, resins, rubbers, fibers. Keratin to modacrylic fibers.

[15] Muzzarelli RA (1993). Biochemical significance of exogenous chitins and chitosans in animals and patients. Carbohydr Polym.

[16] Hempel U, Reinstorf A, Poppe M, Fischer U, Gelinsky M, Pompe W (2004). Proliferation and differentiation of osteoblasts on Biocement D modified with collagen type I and citric acid. J Biomed Mater Res B.

[17] Queffélec CM, Petit M, Janvier P, Knight DA, Bujoli B (2012). Surface modification using phosphonic acids and esters. Chem Rev..

[18] Pascaud P, Gras P, Coppel Y, Rey C, Sarda SP (2013). Interaction between a bisphosphonate, tiludronate, and biomimetic nanocrystalline apatites. Langmuir.

[19] Nejadnik MR, Yang X, Bongio M, Alghamdi HS, Van den Beucken JJ, Huysmans MC (2014). Self-healing hybrid nanocomposites consisting of bisphosphonated hyaluronan and calcium phosphate nanoparticles. Biomaterials.

[20] Wang L, Zhang M, Yang Z, Xu B (2006). The first pamidronate containing polymer and copolymer. Chem Commun.

[21] Cahyanto A, Tsuru K, Ishikawa K (2013). Carbonate apatite formation during the setting reaction of apatite cement. Adv Bioceram Porous Ceram.

[22] Zuo Y, Yang F, Wolke JG, Li Y, Jansen JA (2010). Incorporation of biodegradable electrospun fibers into calcium phosphate cement for bone regeneration. Acta Biomater.

[23] Carey LE, Xu HH, Simon CG, Takagi S, Chow LC (2005). Premixed rapid-setting calcium phosphate composites for bone repair. Biomaterials.

[24] Khairoun I, Boltong M, Driessens F, Planell J (1997). Effect of calcium carbonate on clinical compliance of apatitic calcium phosphate bone cement. J Biomed Mater Res.

[25] Bohner M (2010). Design of ceramic-based cements and putties for bone graft substitution. Eur Cell Mater..

[26] Rau J, Generosi A, Smirnov V, Ferro D, Albertini VR, Barinov S (2008). Energy dispersive X-ray diffraction study of phase development during hardening of calcium phosphate bone cements with addition of chitosan. Acta Biomater.

[27] Li D, Fan H, Zhu X, Tan Y, Xiao W, Lu J (2007). Controllable release of salmon-calcitonin in injectable calcium phosphate cement modified by chitosan oligosaccharide and collagen polypeptide. J Mater Sci Mater Med.

[28] Takagi S, Chow LC, Hirayama S, Sugawara A (2003). Premixed calcium–phosphate cement pastes. J Biomed Mater Res B.

[29] Kai D, Li D, Zhu X, Zhang L, Fan H, Zhang X (2009). Addition of sodium hyaluronate and the effect on performance of the injectable calcium phosphate cement. J Mater Sci Mater Med.

[30] Panzavolta S, Torricelli P, Bracci B, Fini M, Bigi A (2009). Alendronate and Pamidronate calcium phosphate bone cements: setting properties and in vitro response of osteoblast and osteoclast cells. J Inorg Biochem.

[31] Sawamura T, Hattori M, Okuyama M, Kondo K (2004). Effects of polysaccharides addition in calcium phosphate cement. Key Engineering Materials.

[32] Ambrosio L, Tanner E (2012). Biomaterials for spinal surgery.

[33] Hannant D, Hughes D, Kelly A, Alford NM, Bailey J (1983). Toughening of cement and other brittle solids with fibres. Philos Trans R Soc A.

[34] Li VC, Mishra DK, Wu H-C (1995). Matrix design for pseudo-strain-hardening fibre reinforced cementitious composites. Mater Struct.

[35] Nelson PK, Li VC, Kamada T (2002). Fracture toughness of microfiber reinforced cement composites. J Mater Civ Eng.

[36] Barralet J, Gaunt T, Wright A, Gibson I, Knowles J (2002). Effect of porosity reduction by compaction on compressive strength and microstructure of calcium phosphate cement. J Biomed Mater Res.

[37] Gbureck U, Spatz K, Thull R (2003). Improvement of mechanical properties of self setting calcium phosphate bone cements mixed with different metal oxides. Materialwiss Werkstofftech.

